# Clinical Outcome of Antibiotic Suppressive Therapy in Patients with a Prosthetic Joint Infection after Hip Replacement

**DOI:** 10.7150/jbji.37262

**Published:** 2019-11-06

**Authors:** Borg Leijtens, Laura Weerwag, Berend Willem Schreurs, Bart-Jan Kullberg, Wim Rijnen

**Affiliations:** 1 Department of Orthopedic Surgery, Radboud University Medical Centre Nijmegen, the Netherlands; 2 Department of Internal Medicine/Infectious Diseases, Radboud University Medical Centre Nijmegen, the Netherlands

**Keywords:** prosthetic joint infection, antibiotic suppressive therapy, hip replacement

## Abstract

**Introduction:** In Specific cases, curative treatment of a prosthetic joint infection (PJI) cannot be accomplished due to the increased risk of major complications after prosthetic joint revision surgery. In these patients, antibiotic suppressive therapy (AST) is often used to control the infection.

**Aim:** To describe the clinical outcome of patients with a PJI after hip replacement treated with AST.

**Methods:** Patients in which AST for PJI was started between 2006 and 2013, were retrospectively included. Follow-up was continued until October 2018. AST has been defined as treatment with oral antibiotic therapy intended to suppress PJI. Treatment was considered successful in patients without reoperation for PJI or death related to PJI during follow-up.

**Results:** Twenty-three patients were included. The most commonly used antibiotics were doxycycline (n=14) and cotrimoxazole (n=6). The mean duration of AST was 38 months (1-151 months). AST was considered successful in 13 patients (56.5%) after a median follow-up of 33 months. AST was least successful in PJI caused by S. *aureus* with 80% failures versus 33% in PJI caused by other microorganisms and in patients who had an antibiotic-free period before the start of AST with 83% failures. Two patients ended AST due to side effects.

**Conclusion*:*** AST can be an alternative treatment in selected patients with a PJI after hip replacement. However, there is a persisting and considerable amount of failures, particularly in PJI caused by S. *aureus* and in patient with an antibiotic-free period before the start of AST.

## Introduction

Total hip replacement (THR) is a very successful surgical procedure improving patients' quality of life by providing pain relief and restoring function. However, a prosthetic joint infection (PJI) is a devastating complication occurring in 1-3% of patients after primary THR and 10-12% after revision procedures 1. PJIs can be classified as early, delayed and late according to the time of symptom development after the index surgery 1-3. Guidelines recommend to treat an early or hematogenous PJI with debridement, antibiotics and implant retention (DAIR). In general, a delayed or late infection is managed with either a one- or two-stage revision procedure combined with antibiotic treatment 4. However, there are patients in which surgical strategies are contraindicated. This may be due to comorbidities, surgical conditions (e.g. poor bone stock) or patients' refusal to undergo surgical therapy. For these patients, antibiotic suppressive therapy (AST) may be an alternative treatment option. The aim of this suppressive treatment is to control clinical manifestations of the infection rather than cure the infection 5.

Previous studies regarding AST in PJI are few. We have found a total of eight studies reporting clinical outcome of PJI patients treated with AST between 1988 and 2017 6-13. These studies included 13-92 patients treated with a variety of suppressive antibiotics, reporting success rates varying from 23% to 86% with a mean follow-up of 2-5 years. These studies on AST give us variable data on AST in mostly inhomogeneous groups with PJI of hip, knee, shoulder and elbow joints, different type, dosage and duration of antibiotic therapy with relatively short follow-up 6-13.

The aim of this study is to describe the clinical outcome of patients with a PJI after hip arthroplasty who received AST. Factors influencing their clinical outcome are investigated. In our hospital, AST is mainly applied after hip arthroplasty. In order to reach a homogenous group, only patients receiving AST after hip arthroplasty are included.

## Materials and Methods

### Study design and population

This retrospective cohort study was performed at the Radboud University Medical Centre in Nijmegen, the Netherlands. All patients with a PJI were discussed in a weekly multidisciplinary meeting with an orthopedic surgeon, an infection disease specialist and a microbiologist. All patients with a PJI in which treatment with AST was started between January 1, 2006 and December 31, 2013 were included. Follow-up was completed until October 31, 2018. PJI was diagnosed according to the MSIS criteria by means of 2 or more tissue cultures demonstrating growth of an identical pathogen or ≥1 cultured virulent micro-organism 14. We also aimed to analyze the difference in success rate between patients treated with AST after DAIR, two-stage revision or PJI diagnosed after diagnostic puncture. The following data were collected from the patient files: general patient characteristics (age, gender, BMI, ASA classification, type of hip prosthesis, comorbidities), number of revision surgeries of the affected joint, time of onset of PJI, symptoms of PJI, causative microorganism, type, dosage and duration of AST and clinical and radiological outcome. We also collected laboratory results at the start of AST, i.e. C-Reactive Protein (CRP), Erythrocyte Sedimentation Rate (ESR) and leucocyte count.

### Antibiotic suppressive therapy

AST was defined as an oral antibiotic therapy without an end date, started with the intention to control the infection where curative treatment seems unachievable. The type and dosage were based on *in vitro* susceptibility of the cultured pathogens. Laboratory monitoring for potential toxicity and adverse events was performed.

### Outcomes

AST was considered to be successful in cases with retention of the prosthesis without clinical relapse of infection at final follow-up. In cases in which follow up had ended due to the death of the patient unrelated to (the treatment of) PJI, AST was considered successful. Failure was defined as death related to PJI or new surgical intervention at prosthesis side due to persistent or recurrent infection.

### Follow-up

Patients were seen at the outpatient clinic by the orthopedic surgeon or the infection specialist, every three months in the first year. The interval increased to yearly if there were no symptoms of PJI or adverse events due to antibiotics. Endpoints were (unrelated) death, re-intervention at prosthesis side due to infection or latest follow-up at outpatient clinic if no event occurred.

### Statistical Analysis

The statistical analysis was performed with IBM SPSS statistics, version 22.0 (IBM Corp., Armonk, N.Y., USA). To describe overall survival without an event, a Kaplan-Meier survival analysis was performed according to an intention-to-treat principle. To assess the association of risk factors, known from previous studies 8-13, with clinical outcome, a univariate logistic regression analysis was performed. In case off missing values these data were deleted from our analysis. P values < .05 were considered to be statistically significant.

All procedures performed in studies involving human participants were in accordance with the ethical standards of the institutional and/or national research committee and with the 1964 Helsinki declaration and its later amendments or comparable ethical standards. The local ethics committee has waived approval for this study.

## Results

A total of 23 patients (16 female) with a mean age of 70 years (range, 40-88 years) at start of their AST were included. Patient characteristics and medical history are shown in Table 1. The majority of the patients (66.7%) had a PJI after previous revision surgery of the hip. The mean number of previous surgical procedures on the affected side was five operations (range 1-9). Indications for AST were surgical complexity with poor bone stock and severe soft tissue injury (29%), patients wish not to be re-operated (13%), poor general medical condition (21%). 9 patients (38%) had a combination of reasons (surgical complexity, poor general medical condition and/or patients wish not to be re-operated).

### Surgical therapy

20 (87.5%) patients underwent surgery before the start of AST; 13 of these patients underwent DAIR of whom 5 patients were treated with gentamycin polymethylmethacrylate beads. During all DAIR operations, modular implant parts were exchanged and rinsing was performed with 3 liters of betadine-saline solution and 3 liters of normal saline. 7 patients underwent partial or total revision for suspected aseptic loosening or periprosthetic fracture with unexpected positive intraoperative cultures. The 3 (12.5%) patients without surgery underwent a sterile puncture of the hip under suspicion of a chronic PJI. Cultures proved a PJI in these patients who were in a poor medical condition/not suitable for operative treatment. The isolated microorganisms are stated in Table 2.

### Antibiotic therapy

All 20 (87.0%) patients who underwent surgery before the start of AST, initially received intended curative antibiotic therapy for a mean duration of 13.4 weeks (range 0.71-29.9 weeks). In 16 of these patients a rifampin-based combination therapy was given after surgery, in the remaining 4 patients AST was started shortly after surgical intervention.

In 14 patients (60.9%), AST was started immediately after the initial treatment. In 6 patients (26.1%) AST was started when they had a clinical relapse of their symptoms after an antibiotic-free period ranging from 3-24 weeks (mean 16 weeks) after their initial treatment. In 3 patients (12.5%) AST was started after positive sterile puncture of the hip, without initial curative therapy. The mean duration of the AST in our patient group was 38 months (range 1-151 months). The type, dosage and duration of the AST are summarized in Table 2.

### Results of AST

**Figure 1:**
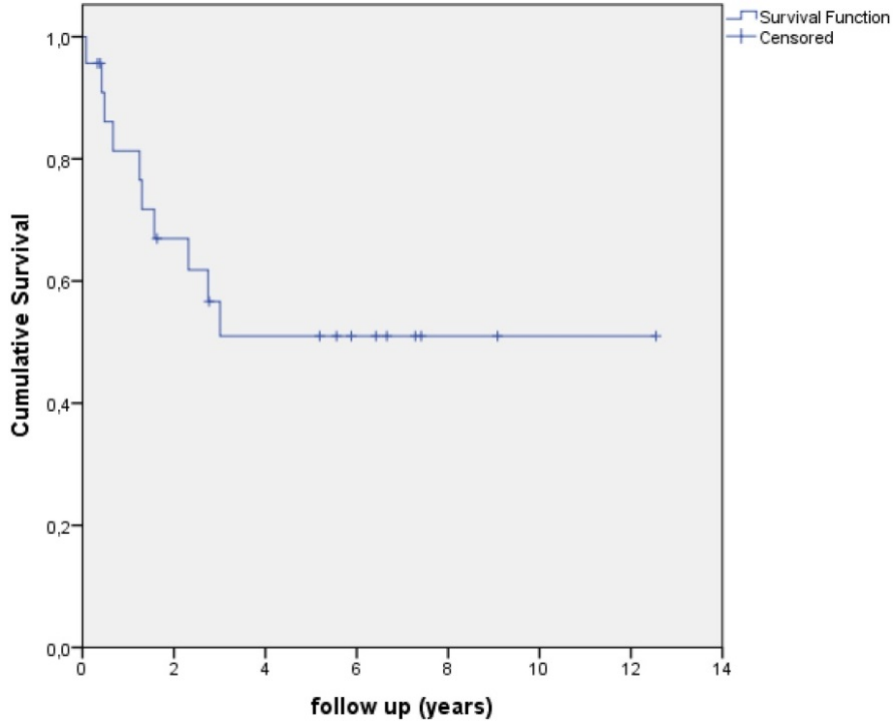
Kaplan-Meier curve showing survival/time without an event in the total group.

At time of final follow-up, AST was considered successful in 13 patients (56.5%) after a median follow-up of 33 months (range 1-151 months). Kaplan-Meier survival analysis estimates a mean symptom-free prosthesis retention period of 82 months (6.9 years) with a 95% confidence interval of 54-111 months. The Kaplan-Meier survival curve is shown in Figure 1. 9 patients (39.1%) had no event during follow-up. 4 patients (17.4%) died of causes unrelated to PJI. 2 of the 13 patients with a successful result ended the AST during follow-up and retained their prosthesis without any sign of infection at final follow-up.

AST failed in 10 patients (43.5%). 7 patients (29.2%) had a relapse of infection with the same micro-organism and 3 patients (13.0%) developed a new infection with a different micro-organism. One of the failures underwent a new surgical intervention (DAIR) after 6 months of AST. In 8 of the failed AST patients a Girdlestone procedure was performed of whom one patient underwent a reimplantation after 3 months. One of the failures underwent a proximal femur resection.

**Figure 2:**
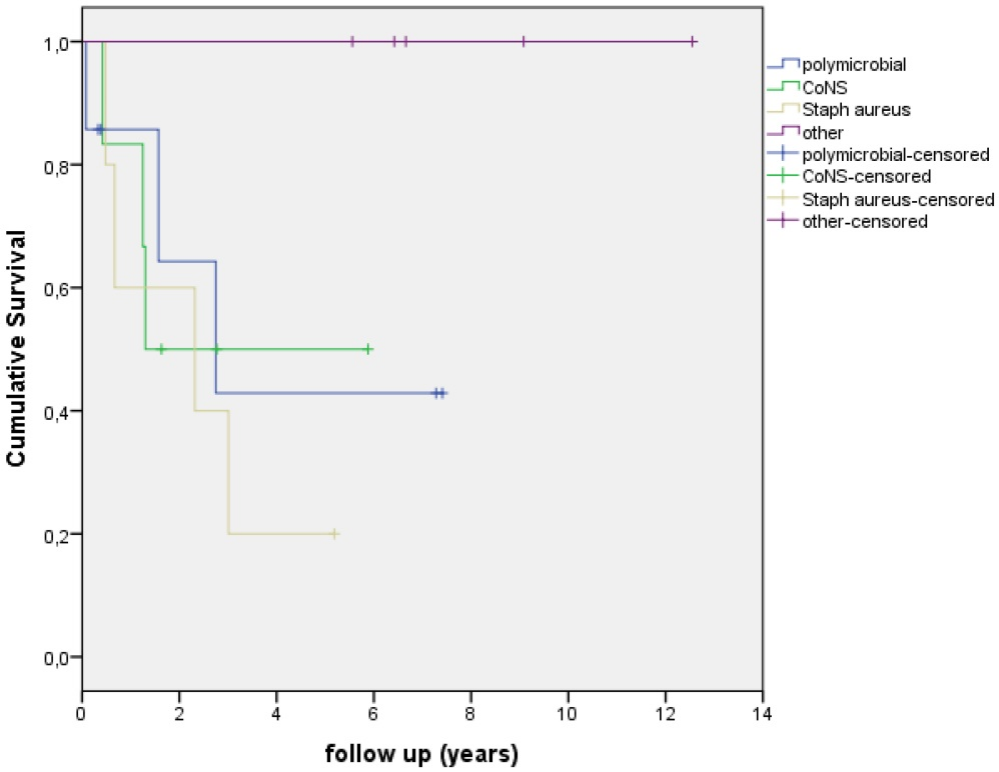
Kaplan-Meier curve showing survival/time without an event with different causative microorganisms.

**Figure 3:**
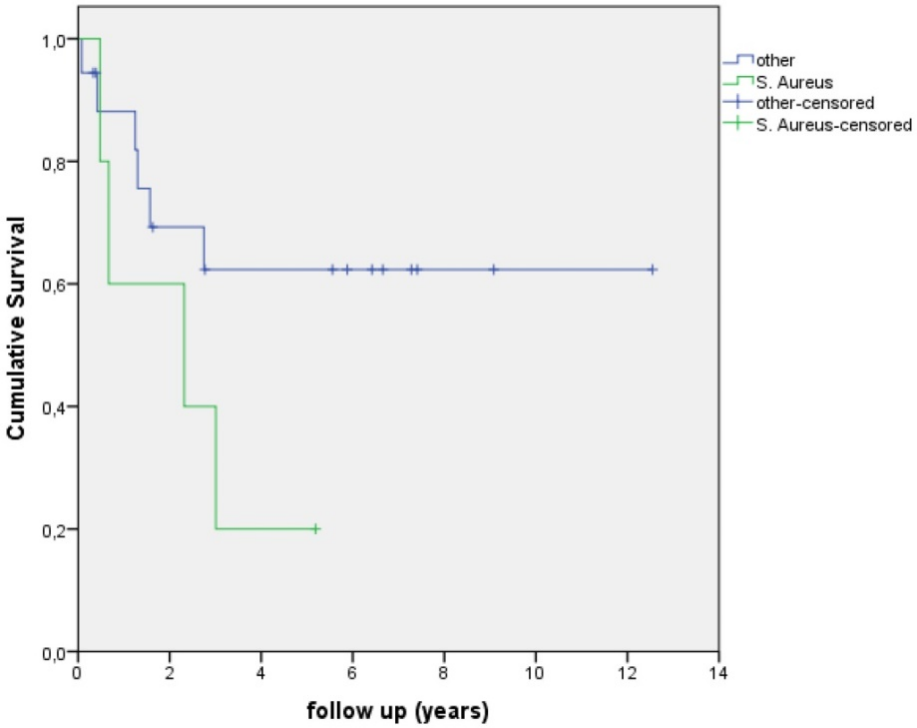
Kaplan-Meier curve showing survival/time without an event, S. aureus vs other causative microorganisms.

**Figure 4:**
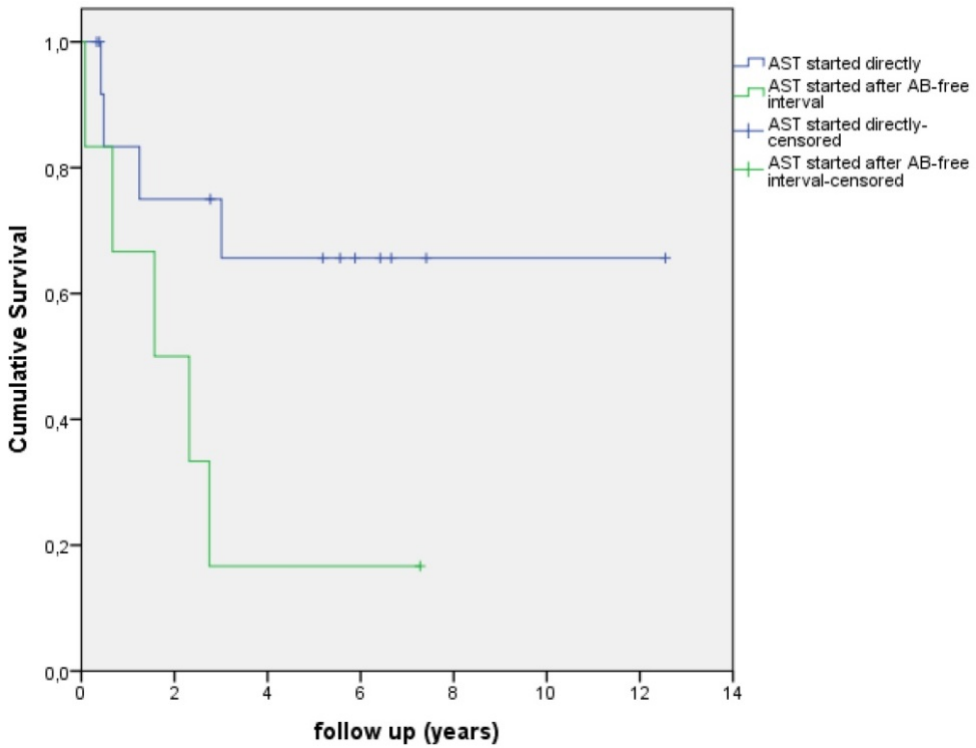
Kaplan-Meier curve showing survival/time without an event, AST started directly vs AST started after AB-free period.

**Figure 5:**
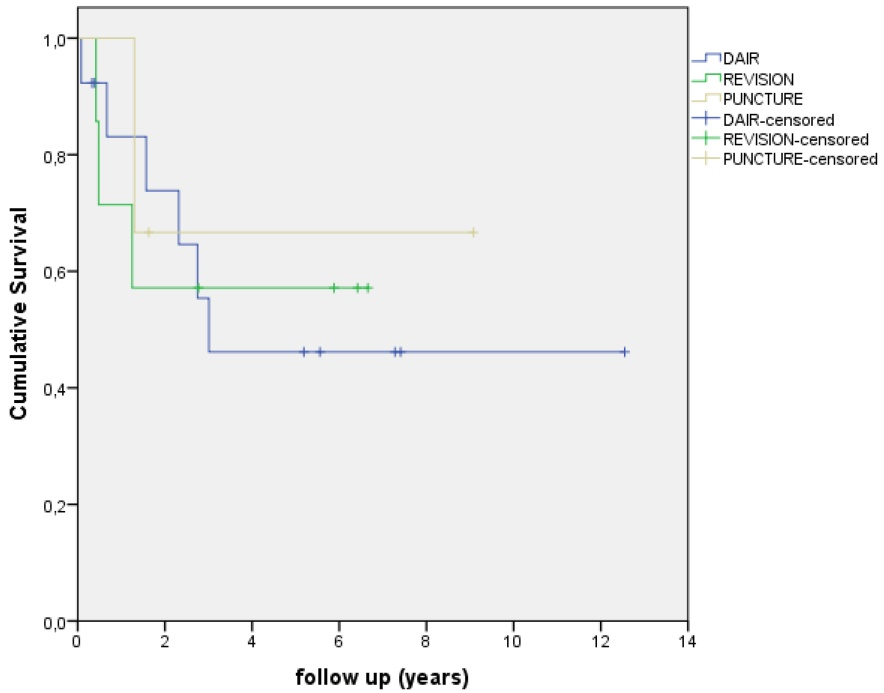
Kaplan-Meier curve showing survival/time without an event, DAIR vs Revision vs Puncture. DAIR: Debridement, Antibiotics and Implant Retention.

In this study 7 patients had a polymicrobial infection (30%), 6 patients had a Coagulase Negative Staphylococci (CoNS) infection (26%), 5 patients had a S. *aureus* infection (22%) and five infections were caused by other microorganisms (22%) (see Table 2). The Kaplan Meier survival curve in Figure 2 shows the survival of different PJI infections. Figure 3 shows the Kaplan Meier survival curve of patients with a PJI with a S. *aureus* infection versus patients with PJI with a different microorganism. 4 out of 5 PJI caused by S. *aureus* failed (80.0%) versus 7 out of 19 failures (37.0%) in the patient group with an infection caused by other microorganisms. This difference however, was not significant (p=0.143). Figure 4 shows the Kaplan Meier survival curve of patients who had an antibiotic-free period before the start of AST versus patients receiving AST directly after intended curative treatment. In 5 out of the 6 patients (83.3%) with an antibiotic-free period, treatment failed, versus 4 out of 14 failures (28.6%) in patients receiving AST directly after surgery (p=0.045). In the three patients with an assumed low-grade infection in which AST was started after puncture, one failure was observed. Figure 5 shows the Kaplan Meier survival curve comparing patients who had a DAIR, one-stage revision or puncture before the start of AST. No significant difference was seen comparing these groups.

Table 3 shows the results of the univariate logistic regression analysis for variables that could possibly be associated with an increased failure rate. None of the variables were significantly associated with an increased risk of failure except for an antibiotic-free period before the initiation of AST.

There were 4 patients in which follow up ended before 6 months of AST. Two of these patients had a clinical relapse of PJI and were marked as failures. One patient died of a cardiac cause unrelated to PJI treatment and the other patient stopped AST due to side effects but retained his prosthesis until final follow up. We separately analyzed patients receiving at least 6 months of AST showing a success rate of 63.2% (12 out of 19 patients).

### Adverse events

6 (26.1%) patients experienced adverse events during AST. 4 of these patients experienced gastrointestinal problems, a rash or itching but could continue suppressive treatment; in 2 of these patients the dosage was reduced, in another patient cotrimoxazole was switched to doxycycline and 1 patient experienced a temporary itching sensation without the need to change or stop the AST. The other 2 patients (8.7%) ended their therapy due to adverse events. 1 of them was suspected to have pleural effusion as an adverse event; further analysis however did not confirm this. The other patient had to end the suppressive therapy due to thrombocytopenia during the use of Doxycycline in combination with TAR syndrome (thrombocytopenia-absent radius syndrome).

## Discussion

AST is suggested to be an alternative treatment in selected patients with a PJI in which further surgical intervention is unattractive. The aim of this study was to describe the clinical outcome of patients treated with AST in PJI after hip arthroplasty.

We found a 56.5% success rate after a median follow-up of 33 months. Considering an estimated mean survival of 82 months (95% CI 54-111), AST appears to be a rational treatment option when curative treatment seems impossible. Success rate in previous studies vary from 23 to 86% 6-13. Table 4 summarizes the results of previous studies reporting on AST 8-13. The presented studies in Table 4 included a wide variety of PJI including infections of total hip, knee, elbow and shoulder prosthesis. Both Siquira et al and Pradier et al found that infection involving the hip joint was associated with a better outcome compared to other PJIs 11, 13. To be able to give a fair impression of what to expect from AST after hip surgery, solely PJI after hip arthroplasty were included.

Siqueira et al was the first study comparing 92 patients treated with AST for PJI with a matched cohort of patients with PJI not receiving AST (ratio 3:1) 11. They found a significant difference in five-year infection-free prosthetic survival rate of 68.5% in the AST group compared to 41.1% in the non-suppression group. Interestingly, they found a greater benefit from AST in patients with a S.* aureus* infection compared to patients with a S.* aureus* infection not treated with AST.

In other previously published literature, several variables associated with a lower chance of survival are described; S.* aureus* infection 8, 9, 10, 12, older age (>85 years), female gender, hypoalbuminemia, presence of a sinus tract 10, tumor prosthesis, higher level of inflammation blood values, rheumatoid arthritis 12, longer initial curative treatment and discontinuation of AST after 2 years 13. We performed a univariate logistic regression analysis for all of these variables as described in Table 3. In accordance with other studies we found a higher failure rate among PJI caused by S. *aureus* (80% versus 37%). However, this difference is not statistically significant. Consistent with the findings of Wouthuyzen et al 12, we found a higher failure rate among patients with higher inflammatory parameter; 60% of the patients with a CRP level ≥ 80ml/L failed, versus 20% of the patients with a CRP level < 80ml/L at the start of AST. In our study, this difference was not statistically significant (p=0.79). Interestingly, we did find one statistically significant variable in this study. We have included 6 patients who had an antibiotic-free period before AST was started. In these patients PJI relapsed after initial curative treatment. In 5 of these patients (83.3%) AST failed versus 4 out of 14 failures (28.6%) in patients receiving AST directly after initial curative treatment (p=0.045). Despite no multivariate regression analysis could be performed, this finding suggests that AST should only be started when followed directly after curative antibiotic treatment and that bacterial load reduction seems essential for AST to be successful. However, the lower success rate in this subgroup of patients could also be explained by the fact that these patients already have shown a relapse of PJI at the start of AST. The inclusion of these patients could possibly explain a lower success rate in our study compared to previous studies described in table 4 8,9,11-13.

### Strengths of this study

This is the first study on AST solely describing patients with a PJI after hip replacement. An intention-intention-to-treat analysis gives a fair impression of what to expect when considering AST in PJI treatment after hip arthroplasty. All patients had a microbiologically proven PJI, the microorganism was susceptible to oral antibiotic therapy, and the type of antibiotic therapy was based on *in vitro* susceptibility of the pathogen. Almost all of our patients used either doxycycline or cotrimoxazole.

The optimal regimen and duration of AST remains uncertain. We generally prescribed doxycycline 100 mg q.d. and cotrimoxazole 480-960 mg q.d. This is half of the recommended dosages of doxycycline 100 mg b.i.d. and cotrimoxazole 960 mg b.i.d., as described in the IDSA guidelines 4. Especially in patients who are treated with AST because of their medical condition, a low dosage of antibiotics is favored because of the possible side effects. Only two patients (8.7%) had to end their antibiotic therapy due to adverse events. Rao et al. showed a similar percentage of 8% 9. In the patient group presented by Segreti et al. 22% percent of the patients showed adverse events 8.

### Study limitations

As in previously performed studies on AST, the retrospective design and low number of patients are the main limitations of our study. Because of the small sample size, a multivariate analysis with Cox-regression analysis could not be performed to assess variables associated with an increased risk of treatment failure. A third limitation of this study is the use of different initial treatment regimens.

## Conclusion

AST may be the only treatment option in patients in which curative treatment with surgical intervention is contraindicated. In addition to previous literature, this study suggests the use of chronic suppressive antibiotics is safe with acceptable outcomes, considering the absence of alternative treatment strategies. When considering the start of AST, one should be aware of a possible decreased success rate among patients who had an antibiotic-free period before the start of AST, patients with high inflammatory parameters and S. *aureus* infections.

Due to the small sample size and inhomogeneous study group in the current and previous studies, we have not been able to identify definite risk factors for failure of AST. Therefore, recommendation on the use of AST in the current international guidelines remains based on the few available data and expert opinions. Ideally, a prospective randomized controlled trial with larger numbers is performed to assess the optimal regimen and safety of antibiotic suppressive treatment. However, this seems an unachievable goal given the exceptional inhomogeneous group of patients and lack of alternative treatment options. Therefore, we emphasize a systematic review of the currently available studies is necessary to facilitate the development of guidelines for routine practice.

## Authors Contributions

Author BL contributed to the article by conception and design of the manuscript, data collection, analysis and interpretation of data and writing of the manuscript.

Author LW contributed to the article conception and design of the manuscript, data collection, initiating in analysis of the data and drafting of the manuscript.

Author BWS contributed to the article by data collection, analysis and interpretation of the data and critically reviewing the manuscript.

Author BJK contributed to the article by conception and design of the manuscript, patient selection, and critically reviewing the manuscript.

Author WR contributed to the article conception and design of the manuscript, by patient selection, analysis and interpretation of data, drafting and critically reviewing the manuscript.

## Figures and Tables

**Table 1:** Patient characteristics of 23 patients with PJI treated with antibiotic suppressive therapy

Characteristics	Number of patients
**Gender**	
Female, n (%)	16 (69.6)
**Age at start AST, years, mean (range)**	70 (40-88)
**BMI, kg/m², mean (range)**	26.6 (16.8-44.8)
**BMI group, n (%)**	
<20	4 (17.4)
20-25	6 (26.1)
25-30	7 (30.4)
30-35	5 (21.7)
>35	1 (4.3)
**ASA score, n (%)**	
1	3 (13.0)
2	12 (52.2)
3	8 (34.7)
**Co-morbidity**	
Cardiovascular	9 (39.1)
Rheumatoid arthritis	1 (4.3)
Diabetes mellitus type 2	2 (8.7)
Malignancy <5 years	3 (13.0)
**Prosthetic joint, n (%)**	
Total hip arthroplasty	21 (91.3)
Hemi arthroplasty	2 (8.7)
**Arthroplasty, n (%)**	
Primary implant	4 (17.4)
Revised implant	19 (82.6)
**Type/onset of PJI, n (%)**	
Early (<3 months)	7 (30.4)
Delayed (3-24 months)	8 (34.7)
Late (>24 months)	8 (34.7)
**Previous PJI, n (%)**	
Yes	9 (39.1)
**DAIR**	
Yes	13 (56.5)
1 x DAIR	8
2 x DAIR	3
3 x DAIR	1
4 x DAIR	1
**Physical examination at time of PJI symptoms, n (%)**	
Fever	5 (21.7)
Sinus tract	5 (21.7)
**Laboratory examination at time of PJI symptoms, n (%)**	
Elevated CRP (>10 mg/l)	19 (82.6)
Elevated total leukocyte count (>11.0 x10^9^/l)	5 (21.7)
**Radiological examination at time of PJI symptoms, n (%)**	
Loosening of the cup	2 (8.7)
Radiolucency of the cup	2 (8.7)
Protrusion of hemi arthroplasty with radiolucency stem	2 (8.7)
Broken osteosynthesis material	1 (4.3)

AST: antibiotic suppressive therapy; BMI: body mass index; ASA: American Society of Anesthesiologists; DAIR: Debridement, Antibiotics and Implant Retention.

**Table 2:** Identified microorganisms and agents, dosages and duration of the used antibiotic suppressive therapy

Patient no.	Microorganism(s)	Type and dosage of AST	Time on AST (months)	Comments/adverse effects	Outcome
1	EC, CoNS	Cotrimoxazole 480 mg q.d	87	No doxycycline because usage of methotrexate	success
2	CoNS	Doxycycline 100 mg q.d	33		failure
3	Clostridium perfringens	Doxycycline 100 mg q.dRifampin 300 mg b.i.d	67	Lower dosage because of nausea and dry mouth	success
4	CoNS, GBS	Cotrimoxazole 480 mg b.i.d Amoxicillin 500 mg t.i.d	19		failure
5	SA, PA	Doxycycline 100 mg q.d	62		success
6	SA	Doxycycline 100 mg q.d	36		failure
7	CA	Cotrimoxazole 480 mg q.d	7	Discontinuation because of possible toxicity (pleural effusion)	success
8	CoNS	Doxycycline 100 mg q.d	33		success
9	CoNS	Cotrimoxazole 960 mg q.d (3 months) followed by doxycycline 100 mg q.d	16	Switch to doxycycline because of nausea	failure
10	CoNS	Doxycycline 100 mg q.d	20	Lower dosage because of nausea	success
11	CoNS	Doxycycline 100 mg q.d	15		failure
12	SA	Ciprofloxacin 500 mg b.i.d	8		failure
13	SA, CoNS	Doxycycline 100 mg q.d	6		failure
14	SA, CoNS	Doxycycline 200 mg q.d	28		failure
15	CoNS, corynebacterium	Doxycycline 100 mg q.d	3	Discontinuation because of thrombocytopenia in patient with TAR syndrome*	success
16	CoNS	Doxycycline 100 mg q.d	69		success
17	Proteus Mirabilis, EF, corynebacterium	Amoxicillin-clavulanate 625 mg t.i.d.	1		failure
18	CoNS	Doxycycline 200 mg q.d	5	Itching sensation but continued treatment	failure
19	Pseudomonas, CoNS	Doxycycline 200 mg q.d Ciprofloxacin 750 mg b.i.d	5		success
20	CoNS, EF	Amoxicillin-clavulanate 625 mg t.i.d	20		success
21	CA	Cotrimoxazole 960 mg q.d	68		success
22	Gram-positive rods	Doxycycline 100 mg q.d.	109		success
23	Serratia Marcescens	Cotrimoxazole 960 mg b.i.d	151		success

EC: Enterobacter clocae; EF: Enterococcus faecalis; CA: Cutibacterium acnes; SA: Staphylococcus aureus; SE: Staphylococcus epidermidis; CoNS: coagulase-negative Staphylococci; GBS: group B Streptococci. q.d: once a day; b.i.d.: twice daily; t.i.d.: three times daily; q.i.d.: four times daily. * TAR syndrome = Thrombocytopenia Absent Radius syndrome

**Table 3:** Univariate regression analysis

Variables	n	Failures (%)	Odds ratio for success (95% CI)	p-valueᵃ
**Gender**				
Male	7	3 (42.9%)		
Female	16	7 (43.8%)	1.04 (0.17-6.23)	0.968
**Age**				
<50	4	1 (25.0%)		
50-70	8	4 (50.0%)		
>70	11	5 (45.5%)		0.713
**ASA score**				
1	3	1 (33.3%)		
2	12	7 (58.3%)		
3	8	2 (25.0%)		0.331
**Sinus tract**				
Not present	19	9 (47.4%)		
Present	4	1 (25.0%)	2.70 (0.24-30.85.)	0.424
**Microbiology**				
CoNS	6	3 (50.0%)		
S. Aureus	5	4 (80.0%)		
Other	5	0 (0.00%)		
Polymicrobial	7	3 (42.9%)		0. 663
**BMI**				
<30	17	8 (47.1%)		
>30	6	2 (33.3%)	1.78 (0.25-12.45)	0.562
**Arthroplasty**				
Primary implant	4	1 (25.0%)		
Revised implant	19	9 (47.4%)	0.37 (0.03-4.23)	0.424
**Type/onset of PJI**				
Early	7	4 (57.1%)		
Delayed	8	3 (37.5%)		
Late	8	3 (37.5%)		0.687
**CRP (3 missing values)**				
<80	10	2 (20.0%)		
≥80	10	6 (60.0%)	6.00 (0.81-44.35)	0.079
**Initial treatment**				
DAIR	13	6 (46.2%)		
Revision	7	3 (42.9%)		
Puncture	3	1 (33.3%)		0.533
**Duration of AST**				
AST <6 months	4	2 (50.0%)		
AST ≥6 months	19	8 (42.1%)	1.375 (0.158-11.937)	0.773
**AB free period**				
Yes	6	5 (83.3)	0.080 (0.007-0.918)	0.042
None	14	4 (28.6)		
Puncture	3	1 (33.3%)		

RA: rheumatoid arthritis; DM: diabetes mellitus; PJI: prosthetic joint infection; ASA: American Society of Anesthesiologists; BMI: body mass index. ᵃ = log rank test

**Table 4:** Previous studies on AST in PJI

Author	Year and Journal of publication	Number of patients	Mean follow-up (years)	Success rate (%)
Goulet et al 6	1988, J. Arthroplasty	19	4.1	63.0
Tsukayama et al 7	1991, J. Orthopedics	13	3.1	23.0
Segreti et al 8	1998, Clin Inf Disease	18	4.1	83.0
Rao et al 9	2003, CORR	36	4.4	86.2
Prendki et al 10	2014, Int J Inf Disease	38	2.0	60.0
Siqueira et al 11	2015, J Bone Joint Surg Am.	92	5.8	68.5
Wouthuyzen-Bakker et al 12	2017, J Bone Joint Infect	21	1.8	67.0
Pradier et al 13	2018, Infection	78	2.8	71.8

## Abbreviations

AST: Antibiotic suppressive therapy
PJI: Prosthetic joint infection
CoNS: Coagulase Negative Staphylococci
S. *aureus*: Staphylococcus aureus
THR: Total hip replacement
MSIS: Musculoskeletal infection society
BMI: Body mass index
ASA: American society of anesthesiologists
CRP: C-reactive protein
ESR: Erythrocyte sedimentation rate
q.d.: once daily
b.i.d.: twice daily
IDSA: Infectious Diseases Society of America
